# Clopidogrel plus Aspirin Use is Associated with Worse Long-Term Outcomes, but Aspirin Use Alone is Safe in Patients with Vasospastic Angina: Results from the VA-Korea Registry, A Prospective Multi-Center Cohort

**DOI:** 10.1038/s41598-019-54390-w

**Published:** 2019-11-28

**Authors:** Seong-Sik Cho, Sang-Ho Jo, Seung Hwan Han, Kwan Yong Lee, Sung-Ho Her, Min-Ho Lee, Won-Woo Seo, Sung Eun Kim, Tae-Hyun Yang, Keun-Ho Park, Jung-Won Suh, Byoung-Kwon Lee, Seung-Woon Rha, Hyeon-Cheol Gwon, Sang Hong Baek

**Affiliations:** 1 Department of Occupational and Environmental Medicine, College of Medicine, Dong-A University, Busan, Korea; 2 Division of Cardiology, Department of Internal Medicine, Hallym University Sacred Heart Hospital, Anyang-si, Gyeonggi-do Korea; 3 Department of Cardiovascular Medicine, Gil Medical Center, Gachon University, Incheon, South Korea; 4 Department of Cardiovascular Medicine, Incheon St. Mary’s Hospital, The Catholic University of Korea, Incheon, South Korea; 5 Department of Cardiovascular Medicine, Daejeon St. Mary’s Hospital, The Catholic University of Korea, Daejeon, South Korea; 6 Department of Cardiovascular Medicine, Soonchunhyang Seoul Hospital, Seoul, South Korea; 7 Department of Cardiovascular Medicine Hallym University Kangdong Hospital, Seoul, South Korea; 8 Department of Cardiovascular Medicine, Busan Paik Hospital, Inje University, Busan, South Korea; 9 Department of Cardiovascular Medicine, Chosun Medical Center, Gwangju, South Korea; 10 Department of Cardiovascular Medicine, Bundang Hospital, Seoul National University, Seongnam, South Korea; 11 Department of Cardiovascular Medicine, Gangnam Severance Hospital, Yonsei University, Seoul, South Korea; 12 Department of Cardiovascular Medicine, Guro Hospital, Korea University, Seoul, South Korea; 13 Department of Cardiovascular Medicine, Samsung Medical Center, Sungkyunkwan University, Seoul, South Korea; 14 Department of Cardiovascular Medicine, Seoul St. Mary’s Hospital, The Catholic University of Korea, Seoul, South Korea

**Keywords:** Interventional cardiology, Translational research

## Abstract

Anti-platelet agents are commonly used in vasospastic angina (VA) patients with comorbidity like coronary artery disease. However, long-term clinical outcomes in the use of aspirin, clopidogrel or the two agents together have rarely been investigated in VA patients. In a prospective study, we enrolled 2960 patients who received coronary angiography and ergonovine provocation test at 11 university hospitals in Korea. Among them, 1838 patients were diagnosed either with definite (n = 680) or intermediate (n = 1212) VA, using the criteria of chest pain, ECG changes and ergonovine provocation test results. They were analyzed according to their use of aspirin, clopidogrel or both, or no anti-platelet agent at all. The primary outcome was time to composite events of death from any cause, acute coronary syndrome (ACS) and symptomatic arrhythmia during a 3-year follow-up. A primary composite outcome was significantly more common in the aspirin plus clopidogrel group, at 10.8% (14/130), as compared with the non-antiplatelet group, at 4.4% (44/1011), (hazard ratio [HR] 2.41, 95% confidence interval [CI], 1.32–4.40, p = 0.004). With regard to the person-time event rate, similar results were shown, with the highest rate in the aspirin plus clopidogrel user at 4.72/1000 person months (95% CI, 2.79–7.96, log-rank test for primary outcome p = 0.016). The person-time event of the ACS rate was also highest in that group, at 2.81 (95% CI, 1.46–5.40, log-rank test for ACS p = 0.116). Kaplan-Meier survival analysis demonstrated poor prognosis in primary outcomes and ACS in aspirin plus clopidogrel users (log-rank test, p = 0.005 and p = 0.0392, respectively). Cox-proportional hazard regression analysis, adjusting for age, sex, history of coronary heart disease, hypertension, diabetes, presence or not of definite spasm, use of calcium channel blocker, demonstrated that the use of aspirin plus clopidogrel is an independent risk for the primary outcome (HR 2.01, CI: 1.07–3.81, p = 0.031). The aspirin-alone group had a similar primary and individual event rate compared to the no-antiplatelet agent group (HR 0.96, CI, 0.59–1.55, p = 0.872). Smokers using aspirin plus clopidogrel had poorer outcomes than non-smokers, with HR 6.36 (CI 2.31–17.54, p = 0.045 for interaction). In conclusion, among VA patients, aspirin plus clopidogrel use is associated with a poor clinical outcome at 3 years, especially in ACS. Aspirin alone appears to be safe for use in those patients.

## Introduction

Antiplatelet agents are commonly used in patients with vasospastic angina (VA) because substantial number of these patients have concomitant coronary atherosclerosis^1^. One study has shown the rate of mixed vasospasm with coronary atherosclerosis is 57.6%^1^. Spasm is thought to occur mainly at an atherosclerotic site with endothelial dysfunction and impaired vasomotion, unlike in a healthy coronary artery^1,2^.

Moreover, some patients with VA may have undergone coronary stenting at a significant stenosis other than at the spasm site in the same coronary artery or in another coronary artery. Accordingly, mono antiplatelet or dual antiplatelet therapy with aspirin and clopidogrel is mandatory in these patients.

Generally, the use of aspirin is discouraged because it could lead to vasoconstriction via the inhibition of prostacyclin^3^. There exist data from *in-vitro* and *in-vivo* studies supporting this concept, but clinical data regarding the use of aspirin in VA are scarce, other than from small observational studies or case reports^2,4,5^. A recent large study has reported aspirin use causing adverse effects in VA patients even at small doses^6^. However, until now there have been no subsequent studies showing adverse effects of aspirin in VA patients.

Clopidogrel is mainly used in patients receiving coronary stenting and is rarely used for patients with VA, irrespective of the presence of significant atherosclerosis. Nevertheless, given the increasing number of patients with ischemic heart disease and subsequent coronary stenting who require clopidogrel therapy, it is an important and clinically relevant issue to examine the safety of clopidogrel usage especially in combination with aspirin in VA patients.

We therefore investigated the long-term safety of aspirin, clopidogrel or both in patients with VA confirmed by coronary angiography (CAG) and an ergonovine provocation test, in a large prospective multicenter cohort in Korea.

## Results

**Figure 1:**
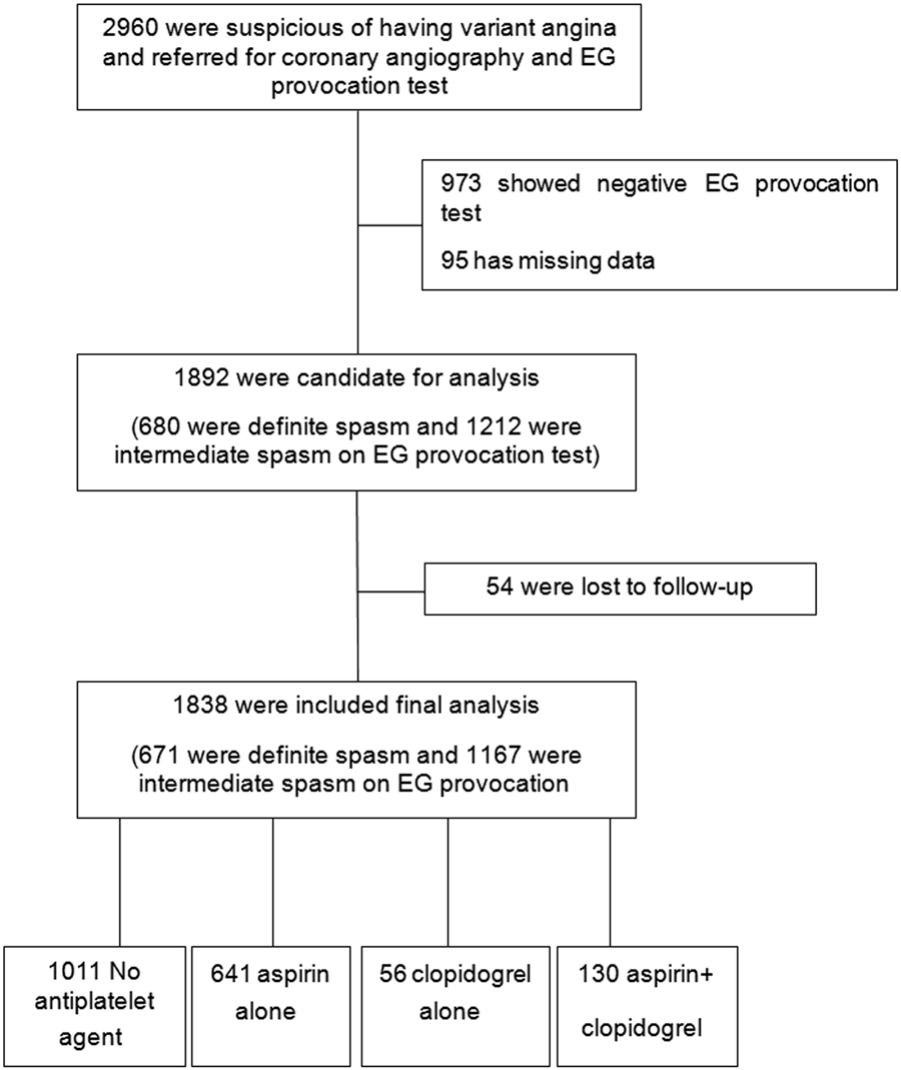
Flow of the study.

We enrolled 2960 patients from 11 hospitals in Korea. From these, 1838 patients with definite/intermediate spasm underwent a final analysis (Fig. 1). The mean follow-up period was 22.63 ± 16.21 months (median 23.90 months, IQR 9.56–23.90).

### Patient demographics and medical history

Mean age ranged from 53.5 to 57 years (with differences in each group). Sex, past medical history, percutaneous coronary intervention (PCI) history, spasm severity, and presence of significant atherosclerosis also differed among the groups. The smoking rate was higher in the clopidogrel and in the clopidogrel plus aspirin group, than in the non-antiplatelet agent group (Table 1).

**Table 1:** Basal clinical characteristics of study participants.

	No antiplatelet agent		Aspirin		Clopidogrel		Aspirin + Clopidogrel		P
	n = 1011		n = 641		n = 56		n = 130		
	Mean	sd	Mean	sd	Mean	sd	Mean	sd	
Age	53.52	11.29	57.23	11.23	57.34	10.70	55.99	10.33	<0.001
BMI	27.78	79.20	25.34	10.60	25.36	5.14	44.13	219.06	0.140
SBP	126.55	39.13	127.60	19.29	127.88	17.81	123.75	18.18	0.629
DBP	84.19	11.85	77.10	12.75	74.94	15.40	76.46	11.73	0.823
Total cholesterol	176.28	35.81	171.45	35.88	181.31	37.11	165.85	38.84	0.002
TG	142.20	102.42	143.27	108.87	153.33	121.13	145.73	101.31	0.893
HDL-Cholesterol	46.87	12.55	46.86	13.67	47.79	9.86	44.39	11.87	0.232
LDL-Cholesterol	104.98	31.02	102.01	31.37	106.60	30.74	98.31	34.10	0.092
eGFR	101.78	37.02	97.21	28.46	95.19	21.89	98.22	32.39	0.039
hsCRP	0.97	6.86	0.74	7.27	0.36	1.11	0.71	3.83	0.883
CKMB	5.59	25.06	5.55	17.63	4.36	5.00	9.93	36.45	0.293
Troponin-I	0.31	2.61	0.75	5.59	0.09	0.36	1.89	7.65	0.023
LV-EF	64.64	6.49	64.49	6.62	64.36	4.07	62.80	8.64	0.057
	**n**	**percent**	**n**	**Percent**	**n**	**percent**	**n**	**percent**	
Sex (male)	590	58.36	412	64.27	39	69.64	100	76.92	<0.001
Smoking	250	24.72	183	28.86	24	42.86	45	34.62	0.005
HTN	320	31.65	294	45.94	22	39.29	61	46.92	<0.001
DM	83	8.22	73	11.42	3	5.36	18	13.85	0.037
Dyslipidemia	160	15.84	98	15.36	20	35.71	22	17.32	0.001
CHD	87	8.61	85	13.30	7	12.5	42	32.31	<0.001
PCI	7	0.69	8	1.25	0	0	25	19.23	<0.001
Definite spasm	326	32.25	253	39.47	23	41.07	69	53.08	<0.001
Atherosclerosis ≥ 50%	35	3.46	55	8.58	8	14.29	36	27.89	<0.001
CKD	3	0.30	2	0.31	0	0.00	0	0.00	0.905
Stains	119	11.90	123	19.65	9	16.67	32	25.81	<0.001
CCBs	162	16.12	152	24.20	9	16.67	31	24.22	<0.001
ARBs	117	11.68	137	21.85	9	16.67	30	24.19	<0.001
ACEIs	9	0.90	15	2.41	2	3.70	6	4.88	0.003
Beta-blockers	59	5.88	54	8.64	6	11.11	18	14.63	0.002
Diuretics	45	4.50	29	4.65	2	3.70	6	4.88	0.987
Anticoagulants	10	1.00	4	0.64	1	1.85	2	1.63	0.629

Sd, standard deviation; BMI, body mass index; SBP, systolic blood pressure; DBP, diastolic blood pressure; TG, Triglyceride; eGFR, estimated glomerular filtration rate by the modification of diet in renal disease study (MDRD) equation; LV-EF, left ventricular ejection fraction; HTN, hypertension; DM, diabetes mellitus; statins, HMG-CoA reductase inhibitors; CCBs, calcium-channel blockers; ARBs, angiotensin-receptor blockers; ACEIs, angiotensin-converting enzyme inhibitors; CKD, chronic kidney Disease; CHD, coronary heart disease; PCI, percutaneous coronary intervention.

### Primary endpoint

Primary endpoints were the composite of occurrences of cardiac death, acute coronary syndrome (ACS), and new-onset symptomatic arrhythmia during a 3-year follow-up.

**Figure 2:**
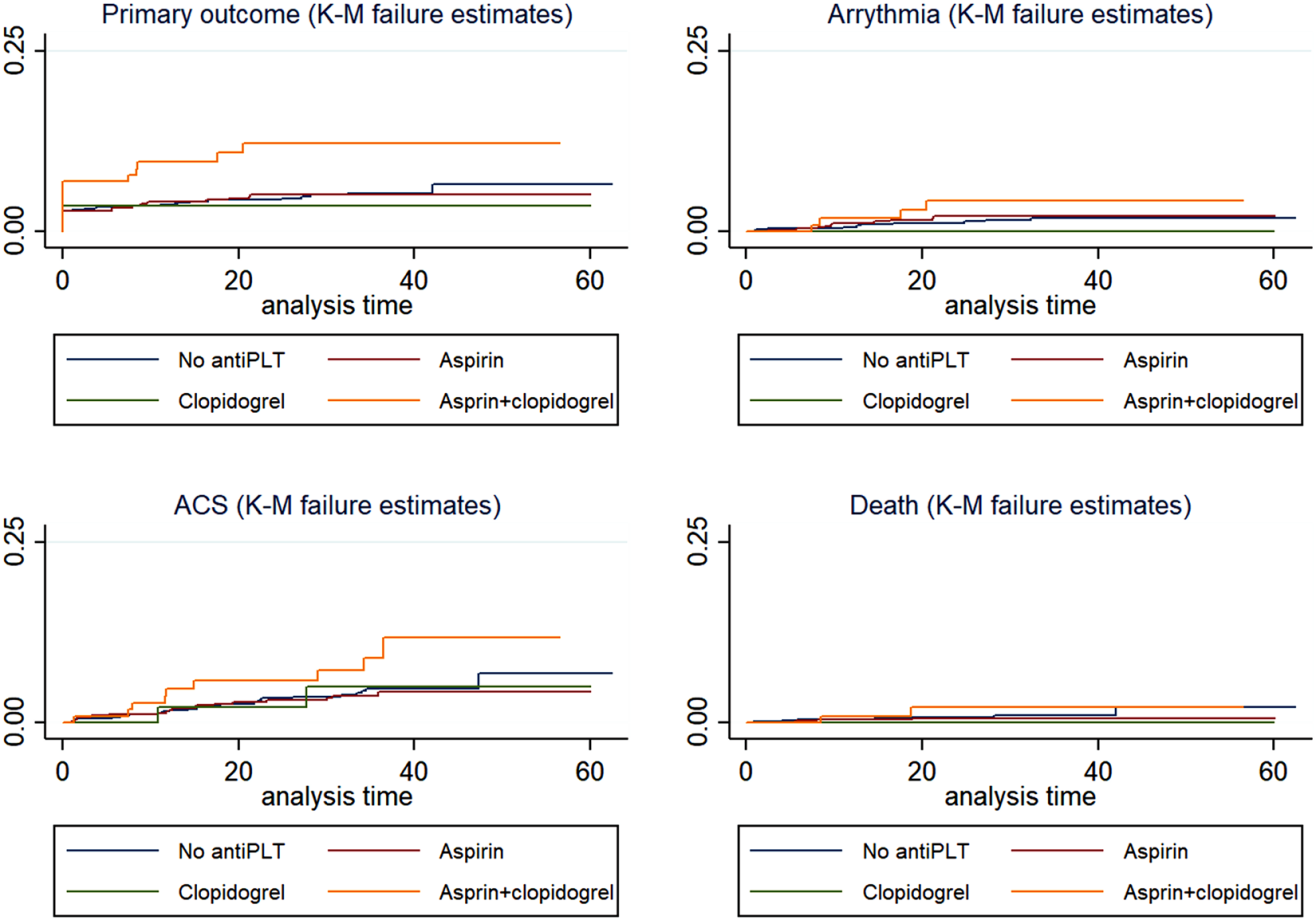
Primary outcomes and individual event comprising primary outcomes according to anti-platelet agent usage with Kaplan-Meier survival curve.

The incidence rate of primary outcomes was significantly higher in patients taking both aspirin and clopidogrel at 10.8% (14/130) compared with non-antiplatelet users, at 4.4% (44/1011) (hazard ratio [HR] 2.41, 95% confidence interval [CI], 1.32–4.40, p = 0.004) (Table 2). With regard to the person-time event rate, there were similar results; with the highest rate occurring in aspirin plus clopidogrel users at 4.72/1000 person months (95% CI, 2.79–7.96, log-rank test for the primary outcome p = 0.016) (Table 2, Fig. 2).

**Table 2:** Incidence rates and Hazard ratios (by uni-variable Cox-proportional hazard model) for primary outcome and components.

	no	Events (%)	Person-month	rate	95% CI		p*	HR	95% CI		P
Primary outcome							0.0155				
No antiplatelet agent	1011	44(4.4)	21265	2.07	1.54	2.78		ref			
Aspirin	641	29(4.5)	15816	1.83	1.27	2.64		0.99	0.62	1.58	0.957
Clopidogrel	56	2(3.6)	1547	1.29	0.32	5.17		0.76	0.18	3.13	0.703
Aspirin + clopidogrel	130	14(10.8)	2968	4.72	2.79	7.96		2.41	1.32	4.40	0.004
Arrhythmia							0.3164				
No antiplatelet agent	1011	11(1.1)	22153	0.5	0.28	0.9		ref			
Aspirin	641	10(1.6)	16368	0.61	0.33	1.14		1.25	0.53	2.94	0.612
Clopidogrel	56	0(0.0)	1620	0							
Aspirin + clopidogrel	130	4(3.1)	3287	1.22	0.46	3.24		2.48	0.79	7.80	0.12
ACS							0.1164				
No antiplatelet agent	1011	28(2.8)	21899	1.28	0.88	1.85		ref			
Aspirin	641	18(2.8)	16258	1.11	0.7	1.76		0.87	0.48	1.57	0.635
Clopidogrel	56	2(3.6)	1639	1.22	0.31	4.88		0.98	0.23	4.12	0.98
Aspirin + clopidogrel	130	9(6.9)	3205	2.81	1.46	5.4		2.19	1.03	4.65	0.04
Death							0.4958				
No antiplatelet agent	1011	7(0.7)	22351	0.31	0.15	0.66		ref			
Aspirin	641	3(0.5)	16555	0.18	0.06	0.56		0.59	0.15	2.28	0.442
Clopidogrel	56	0(0.0)	1673	0							
Aspirin + clopidogrel	130	2(1.5)	3370	0.59	0.15	2.37		1.95	0.4	9.37	0.407

rate: event/1000 person-month; HR, hazard ratio; CI, confidence interval;

P*: p values of log-rank test.

The ACS rate was also higher in the aspirin plus clopidogrel group, at 6.9% (9/130) than it was in the non-antiplatelet group, at 2.8% (28/1011), (HR 2.19, 95% CI, 1.03–4.65, p = 0.04). The person-time event of the ACS rate was highest in that group, at 2.81 (95% CI, 1.46–5.40, log-rank test for ACS p = 0.116) (Table 2). Total death and arrhythmia rates did not differ among the groups (Table 2).

The aspirin-alone group showed a similar rate of occurrence of primary and individual events as the non-antiplatelet group. The aspirin dose used in this cohort was mainly 100 mg (98.3%, 754/767) (200 mg, 1.3% and 300 mg, 0.4%) and there was no difference in events rates according to dose.

We also found the use of aspirin plus clopidogrel to be an independent risk factor for primary endpoints by Cox proportional hazard regression analysis including age, sex, history of coronary heart disease (CHD, CHD was defined as a composite of history of angina with ischemic evidence, coronary artery disease with medical management after coronary angiography, PCI history and CABG history), as well as smoking, hypertension, diabetes, dyslipidemia, spasm severity, whether the patient was taking a calcium channel blocker (CCB) or not, and type of antiplatelet agents (HR 2.01, 95% CI, 1.07–3.81, p = 0.031) (Table 3). Dyslipidemia (HR 1.89, 95% CI, 1.17–3.06, p = 0.010) and definite spasm (HR 1.55, 95% CI, 1.00–2.39, p = 0.048) were also independent risks (Table 3).

**Table 3:** Result of Cox proportional hazard regression for primary outcome.

	HR	95% CI		p
Age	1.00	0.98	1.02	0.821
Female	0.96	0.57	1.59	0.876
History of CHD	1.48	0.84	2.61	0.170
Smoking	1.17	0.71	1.93	0.532
HTN	0.72	0.45	1.15	0.170
DM	1.16	0.59	2.30	0.657
Dyslipidemia	1.95	1.20	3.17	0.007
eGFR	1.00	0.95	1.00	0.320
**Antiplatelet agents**				
No antiplatelet agent	reference			
Aspirin	1.00	0.61	1.62	0.987
Clopidogrel	0.62	0.15	2.60	0.516
Aspirin + clopidogrel	2.15	1.13	4.07	0.019
Definite spasm	1.47	0.95	2.29	0.085
Calcium Channel blocker	0.96	0.46	2.00	0.906

HR, Hazard ratio; CI, confidence interval; CHD, coronary heart disease; HTN, hypertension; DM, diabetes mellitus; PCI, percutaneous coronary intervention.

*Primary outcome includes death, acute coronary syndrome, and arrhythmia.

We performed additional Cox multivariate analysis including troponin I and atherosclerosis ≥50% for balancing the risk between the groups (Supplementary Table 1). In this analysis, we found the same trend towards an incremental risk of clinical outcomes in the aspirin plus clopidogrel group with HR 1.84 (95% CI, 0.89–3.79, p = 0.100) when compared to the non-antiplatelet agents group. By contrast, the aspirin-alone and clopidogrel-alone groups did not show a difference compared with the non-antiplatelet group (Supplementary Table 1). The same Cox multivariate analysis of patients without a history of PCI also demonstrated the increased risk of a primary outcome in dual antiplatelet agent users, with HR 1.65 (95% CI, 0.71–3.84, p = 0.243) (Supplementary Table 2), but with a lack of statistical significance.

### Subgroup analyses

Major subgroup analyses were performed according to risk group; i.e. sex, age, smoking status, hypertension, diabetes, dyslipidemia, CHD history, PCI history, spasm severity by EG provocation test, and combined atherosclerosis. The primary endpoint rates in the subgroups are given in Table 4 with the Supplementary Figure. This analysis was conducted in order to compare aspirin plus clopidogrel users with non-antiplatelet users; the findings showed that aspirin plus clopidogrel caused consistent harm except in the case of the group with a positive history of PCI. We found a statistically significant effect of smoking; smokers using aspirin plus clopidogrel had poorer outcomes than non-smokers with HR 6.35, CI 2.30–17.52, p = 0.025 for interaction.

**Table 4:** Subgroup analysis and interaction between aspirin plus clopidogrel and no-antiplatelet agents group.

		No antiplatelet agent	Aspirin + clopidogrel	HR**	95% CI		p for interaction
		N = 1011	N = 130				
		Event*/n (%)	Event*/n (%)				
Over all		44/1011 (4.4%)	14/130 (10.1%)	2.41	1.32	4.40	
Sex							0.2349
	Male	22/590 (3.7%)	12/100 (12.0%)	3.20	1.58	6.48	
	Female	22/421 (5.2%)	2/30 (6.7%)	1.23	0.29	5.23	
Age							0.6850
	<65	35/854 (4.1%)	9/100 (9.0%)	2.15	1.04	4.49	
	≥65	9/157 (5.7%)	5/30 (16.7%)	2.67	0.9	7.99	
Smoking							0.0250
	Non smoker	37/742 (5.0%)	6/85 (7.1%)	1.36	0.58	3.23	
	Current smoker	7/250 (2.8%)	8/45 (17.8%)	6.35	2.30	17.52	
HTN							0.5780
	No	31/691 (4.5%)	9/69 (13.0%)	2.85	1.36	5.99	
	Yes	13/320 (4.1%)	5/61 (8.2%)	1.98	0.70	5.55	
DM							0.8464
	No	40/927 (4.3%)	12/112 (10.7%)	2.45	1.29	4.67	
	Yes	4/83 (4.8%)	2/18 (11.1%)	1.76	0.32	9.72	
Dyslipidemia							0.8316
	No	33/850 (3.9%)	10/105 (9.5%)	2.31	1.14	4.70	
	Yes	11/160 (6.9%)	4/22 (18.2%)	2.51	0.79	7.93	
CHD							0.4692
	No	39/923 (4.2%)	7/88 (8.0%)	1.81	0.81	4.04	
	Yes	5/87 (5.8%)	7/42 (16.7%)	3.07	0.97	9.68	
History of PCI							0.2552
	No	42/1004 (4.2%)	9/105 (8.6%)	1.96	0.95	4.02	
	Yes	2/7 (28.6%)	5/25 (20.0%)	0.72	0.14	3.73	
Spasm severity							0.7711
	Intermediate	24/685 (3.5%)	4/61 (6.6%)	1.87	0.65	5.37	
	Definite	20/326 (6.1%)	10/69 (14.5%)	2.26	1.06	4.82	
Atherosclerosis							0.7397
	<50%	42/976 (4.3%)	10/94 (10.6%)	2.45	1.23	4.89	
	≥50%	2/35 (5.1%)	4/46 (11.1%)	1.90	0.35	10.37	

HR, Hazard ratio; CI, confidence interval; HTN, hypertension; DM, diabetes mellitus; CHD, coronary heart disease; PCI, percutaneous coronary intervention Event*: the number of incidence cases of the primary outcome; HR**: hazard ratio of the patients with aspirin and clopidogrel compared to patients with no antiplatelet agent.

## Discussions

The VA-Korea registry is a large nation-wide registry composed of patients receiving EG provocation testing on suspicion of VA. The present study comprised patients who showed definite or intermediate EG provocation test results. The aim of the study was to investigate the clinical effect of aspirin and clopidogrel on VA patients. We concluded that the composite risk of death, ACS, and arrhythmia, as assessed in a time-to-event analysis, was significantly higher in those patients who received aspirin plus clopidogrel than in those who received no antiplatelet agent. In particular, the risk of ACS was markedly higher in the aspirin plus clopidogrel users than in the non-antiplatelet, aspirin-only, and clopidogrel-only users.

The higher rate of these endpoints was consistent across multiple subgroups, showing that aspirin plus clopidogrel could aggravate cardiovascular outcomes, particularly ACS, in a wide range of patients regardless of traditional risk factors, i.e. CHD history, PCI history, spasm severity or coronary atherosclerosis ≥ 50%. Aspirin plus clopidogrel had adverse effects in current smokers with a significant p for interaction of 0.025.

Another important finding was that aspirin use alone was not associated with poorer clinical outcomes. The rate of total composite endpoint and of each clinical outcome did not differ between the aspirin group and the no-antiplatelet agents group.

Our study is the first to establish the potential for adverse effects from the combination of aspirin plus clopidogrel in VA patients, with long-term follow-up. Additionally, our study demonstrated the apparent safety of low dose aspirin in VA patients, differentiating it from several recent studies.

### Clinical implications

Clopidogrel is rarely used for VA patients, but under the current guidelines, is likely to be used in combination with aspirin for those patients who have received stents or for the purpose of secondary prevention for those who have atherosclerotic cardiovascular disease. Therefore, our study results have significance for clinical practice in view of the increasing prevalence of coronary intervention for coronary disease. Our findings may also be significant given that there have been reports in the literature that stented segments or adjacent segments are developing spasticity, and this may need to be the subject of further investigation^7,8^.

Our results suggest that VA patients who have stented segments or who have experienced ACS and who should be receiving dual antiplatelet agents, might also require concomitant vasodilatory drugs even if coronary revascularization has been established, because of the possibilities of vasospasm occurring at an adjacent segment.

ACS was a main component of poor clinical outcomes in VA patients taking aspirin plus clopidogrel in our study. The possible explanation is that ACS could occur in VA patients due to the tendency of aspirin plus clopidogrel to aggravate vasospasm. It is noteworthy that the primary endpoint is clearly evident in the subgroup of current smokers, and smoking is a well-known strong risk factor for vasospastic angina^8^. Therefore, the use of combination of aspirin and clopidogrel would be more strongly associated with poor clinical outcomes in the VA smoker group. Definite spasm was also an independent risk factor for the primary endpoint by the Cox proportional hazard model. So careful consideration should be taken in using aspirin plus clopidogrel in current smokers with severe spasm evident by CAG.

Historically, the use of low dose aspirin is regarded as safe in VA patients^9^. However, some registries have reported contrasting results from ours, and have reported that aspirin could be harmful even at low-doses^6,10^. The theoretical rationale for this is that aspirin decrease tissue prostcycline levels and leads to coronary vasoconstriction.

Our study differs from these studies in that our study population was larger by a factor of 2.4^6^. Moreover, each aspirin and no-antiplatelet group was also twice the size of that study^6^. Another distinction is outcome measures. Clinical outcomes of the above study included revascularization, re-hospitalization requiring CAG, and medication change due to recurrent angina. These are ‘soft’ end points and not directly related with VA per se. With regard to the ‘hard’ clinical outcomes of cardiac death, all-cause death, myocardial infarction and re-hospitalization requiring medical intervention, there was no difference between aspirin and non-users. Only re-hospitalization requiring CAG was significantly higher in aspirin group in that study^6^. This suggested that the use of aspirin appears safe in long term follow-up in VA patients, which is in accordance with our study results.

Our results showed that, within a larger population, the use of aspirin 100 mg/day in VA patients is safe.

### Mechanism

Platelet inhibition is the mainstay of therapy for ACS and severe atherosclerotic lesions. These agents have been reported to have some pleiotropic effects which could play a role in vascular inflammation, atherosclerosis, pathogenesis of plaque rupture, and endothelial oxide bioavailability^11^. Nitric oxide (NO) synthesis (NOS) and NO bio-availability possibly account for the poor clinical outcomes using aspirin plus clopidogrel in VA patients.

One *in-vitro* study investigating the effect of aspirin and clopidogrel on NOS demonstrated that the chronic clopidogrel treatment suppresses both basal platelet NOS and beta-adrenergic receptor-stimulated NOS. The study concluded that aspirin also suppressed beta-adrenergic receptor-stimulated NOS^12^. Therefore this combination might lead, at least additively, to negative effects on platelet NOS, resulting in vasoconstriction in the coronary arteries.

However, another study has demonstrated the apparently contradictory result of favoring clopidogrel’s effect on the endothelial NO bioavailability in coronary artery disease patients^11^. The patients in this study had established coronary artery disease and thus did not match well to the patients with VA or those with minimal stenosis or normal coronary arteries. So their conclusions do not apply to our study.

Similarly, in an animal study assessing clopidogrel’s effect on reperfusion injury, clopidogrel decreased the level of NO in ischemic tissues, which might impact poorly on ischemic tissues^13^. However, clopidogrel has not been reported to have an influence on prostacyclin^14^.

Nevertheless, we can assume that the clopidogrel could show reduced efficacy in inhibiting platelet function during periods of endothelial dysfunction, like VA, as is evident from a study in which high on-treatment platelet reactivity was observed despite using clopidogrel in an endothelial dysfunction state, which was evidenced by flow-mediated dilation^15^.

Other plausible mechanism is that the dual antiplatelet therapy might not be sufficient in preventing stent thrombosis due to poor compliance or low efficacy of antiplatelet agents in specific patient who had genetic dysfunction in exerting antiplatelet activity. Although exact data on number of stented patients let alone stent thrombosis rate were not recorded and not provided in our study, stent thrombosis is one of possible explanation of poor clinical outcome in patients who had low antiplatelet benefit with dual antiplatelet therapy.

Dual antiplatelet agents group had multiple combined disease and this lead to high on-treatment platelet reactivity (HTPR)^16^. HTPR, especially to clopidogrel is reported to be associated with more ischemic event and this can also be an explanation for poor clinical outcome in that group^17^.

### Limitations

Our study has some limitations, as follows.

First, there were only 130 patients in the aspirin plus clopidogrel group. The small size of that group may have given rise to bias and chance. Therefore, we have attempted to control the bias with several statistical methods to adjust confounding factors. (More details on this follow).

Nevertheless, our results are significant because this group of patients could hardly be included in VA study let alone the clinical impact of our study calling for caution in treating VA patients who require dual antiplatelet agents.

Second, it is possible that patients on both aspirin and clopidogrel were more likely to receive PCI and have CHD, and thus were more likely to experience adverse clinical events including ACS. However, due to the small sample size of the aspirin plus clopidogrel group, we did not perform propensity matching analysis to balance the baseline characteristics among the groups and this may have influenced the results. Instead, we tried to overcome this shortcoming by multivariate Cox-regression analysis to adjust the confounding factors and sub-group analysis. In addition to this, we performed other multivariate Cox regression analysis which included additional confounders like higher Troponin I, CHD, PCI, and definite spasm. To avoid the potential bias of selecting patients at high risk in the dual antiplatelet agent group, we performed multiple Cox regression analysis only in those patients with no history of PCI. Taken together, we observed a consistent finding of poorer primary outcomes in dual antiplatelet agent users among VA patients, which could support our hypothesis. Although the hazard ratio was 1.65 in dual antiplatelet group as compared to non-antiplatelet user in additional multivariate Cox regression analyses excluding patients with history of PCI, the failure of statistical significance might be attributed to the small sample size of this group and many co-variables (see Supplementary Tables 1 and 2). Subgroup analysis consistently demonstrated poorer outcomes in the dual antiplatelet group. Therefore, we consider dual antiplatelet use still appears to be associated with poor clinical outcomes in VA patients.

Third, we did not assess other clinical outcomes such as episodes of angina, re-admission for angina, and revascularization for the purposes of comparison with other outcomes in our study. More specifically, we did not separate acute myocardial infarction and unstable angina from ACS in the analysis.

Fourth, we do not have the exact maintenance data of drugs and their adherence rates. We suspect that the poor compliance to aspirin might have affected on failure of lowering ACS rate in only aspirin group. Nonetheless, although we do not have the data on aspirin compliance, it is still possible that aspirin alone is neutral in VA patients considering the relatively large sample size of aspirin alone group and consistent results in various analysis in this study.

Fifth, the reason for using both aspirin and clopidogrel could mainly be attributed to the history of PCI and CHD of the patients, occurring at a rate of 19.2% and 32.3%, respectively, in the aspirin plus clopidogrel group. Although this rate is highest of the four groups, it is not entirely clear why the remaining patients in that group used this combination of drugs. We can assume that the patients might have a high atherosclerotic burden or atrial fibrillation and so on. In practice, in Korea, physicians sometimes use dual antiplatelet agents for stroke prevention other than warfarin or the new oral anticoagulants (NOACs); they did so especially in the period prior to the introduction of NOACs, the time in which this study was performed^18^.

Sixth, we do not have bleeding data which can be a major outcome parameter. We did not take into account bleeding as a study endpoint at the study designing stage because we focused more on ischemic events in this population.

Nonetheless, our study has an advantage in being large scale and having long-term follow-up data, including hard clinical outcomes. Our study is also notable in being the first to draw attention to the adverse effects of aspirin plus clopidogrel in VA patients and show evidence of the safety of low dose aspirin in VA patients on a large scale, in the current era in which antiplatelet agents are being used with increasing frequency with an associated increment of combined disease.

## Methods

### Patients

VA-Korea (Variant angina Korea) registry is a nation-wide prospective multicenter registry which enrolled patients with chest pain suggestive of VA who received CAG and an ergonovine provocation test^1^.

Adults aged 18 or over were candidates for enrollment. Patients who had a normal or minimal (<50% luminal diameter narrowing) coronary atherosclerotic stenosis at the baseline CAG were eligible. Patients having malignancy, end stage renal disease on dialysis, inflammatory disease, and catheter-induce spasm at baseline CAG were excluded.

A total of 2960 patients were registered consecutively from May 2010 to June 2015 in 11 tertiary hospitals in Korea having undergone high volume CAG and percutaneous coronary intervention (PCI). Of these, 1892 patients had positive results (680 definite and 1212 intermediate) in their provocation tests. Fifty-four patients were lost to follow-up; thus 1838 patients were included in the final analysis (Fig. 1). Patients with positive results on EG provocation test and spontaneous vasospasm received medical treatment including calcium channel blockers and other vasodilators during follow-up. Patients were divided into four groups according to their differing use of aspirin and clopidogrel during follow-up as; no-antiplatelet agents, aspirin only, clopidogrel only, and aspirin plus clopidogrel. They were followed up for 3 years and monitored during this time for clinical events.

The study protocol complied with the Declaration of Helsinki and was approved by the institutional review boards of Hallym University Hospital (Hallym University Sacred Heart Hospital IRB) and each participating hospital. All procedures and methods were undertaken in accordance with the guidelines of each hospital. All patients gave written informed consent.

### Primary endpoints

The primary endpoints were the composite of death from any cause, acute coronary syndrome (ACS), and new-onset symptomatic arrhythmia during the 3-year follow-up period.

Acute coronary syndrome (ACS) was defined as recurrent or continuous ischemic chest pain lasting more than 20 minutes with ischemic evidence of ECG changes and/or elevation of cardiac markers, including myocardial infarction. Definition of ischemic ECG changes were: an ST elevation of 0.1 mV or more, an ST depression of 0.1 mV or more, T-wave inversion, or a new appearance of left bundle branch block (LBBB) as recorded in at least two contiguous leads on the 12-lead ECG^19^.

We defined a new-onset symptomatic arrhythmia as atrial or ventricular tachycardia/fibrillation, symptomatic premature beats, sick-sinus rhythm, or atrioventricular block which occurred for the first time^1,20^. ECG was routinely checked during the regular follow-up and during emergency visits to the out-patient clinic or emergency department (ED) of the hospital. Holter monitoring for 24-hours was performed in patients with suspicious arrhythmic symptoms. ED revisits due to occurrence of a primary endpoint or of any discomfort were also reported as soon as possible.

An individual adverse event was analyzed as a secondary endpoint. All adverse events of interest were confirmed through source document review, including medical records as well as telephone interviews, and were adjudicated by the local events committee.

### Coronary angiography and ergonovine provocation test

All the patients received standard routine baseline CAG after cessation of vasoactive drugs like calcium channel blockers (CCBs) and nitrate for 48 hours prior to CAG.

All participating hospitals used the same EG provocation test protocol using the same EG administration route and serial dose.

After confirmation by physicians that there was no significant atherosclerotic coronary narrowing from the baseline CAG, EG provocation test was performed starting with the right coronary artery (RCA). If the RCA was intact, the left coronary artery (LCA) was then tested. The EG was mixed with saline and administered by intracoronary bolus (IC) injection over 2–3 minutes. If no narrowing was found after the first dose of EG on RCA angiogram, the dose was escalated from 10 μg (E1) to 20 μg (E2) and 40 μg (E3) sequentially. CAG was performed 1–2 minutes after completion of EG injection in the same projection and compared with the vessel diameter of the baseline RCA.

If coronary spasm was not provoked after testing the RCA, EG provocation on LAD followed with incremental doses of 20 μg (E1), 40 μg (E2), and 60 μg (E3) in the same manner. Once vasospasm was provoked, a bolus of intracoronary nitroglycerine of 200 μg was injected.

After the provocation test, irrespective of the test result, intracoronary (IC) nitrate (200 μg) was injected and the response was observed.

We followed provocation methods from the JCS guideline for diagnosis and treatment of patients with vasospastic angina^19^.

The CAG result was analyzed for each coronary artery and each arterial segment.

Angiographic findings were analyzed on-line or off-line by a dedicated quantitative coronary angiography program or by manual assessment by investigators in each hospital who were not involved in the study. In addition, investigators at the core laboratory of Seoul St. Mary’s Hospital in South Korea, confirmed (blindly) the angiographic data off-line by visual assessment^1^. Meaningful atherosclerosis in each coronary artery was defined as luminal diameter narrowing ≥50%.

### Adjudication of ergonovine provocation test results

Definite (positive) vasospastic angina (VSA) was defined as a total (100%) or subtotal (>90% luminal diameter narrowing) occlusion of the index coronary artery accompanied by ischemic symptoms and/or electrocardiographic (ECG) changes. (201 (1) An ischemic ECG change was defined as an ST segment elevation or depression >0.1 mV or a negative U-wave in at least two contiguous leads^1^. An intermediate result was defined as a patient with 50–90% luminal narrowing with or without ischemic symptoms and/or ECG changes.

We defined negative results as both LCA and RCA EG provocation tests with <50% luminal narrowing without ischemic symptoms nor ECG changes.

### Statistical analysis

Continuous variables were expressed as mean and standard deviations and mean differences between the groups were estimated by the analysis of variance (ANOVA). Categorical variables were demonstrated with numbers and percentages. Each incident’s rate and 95% confident interval (CI) was demonstrated according to the use of anti-platelet agents. Events per 1,000 person month was displayed for estimating the incidence rate of primary endpoint, ACS, arrhythmia, and total death. Kaplan-Meier curves were presented for survival estimation. The log-rank test was conducted to compare the survival among different groups by usage of anti-platelet agent. In addition, the Cox proportional hazard regressions were conducted to estimate the survival difference among groups. The hazard ratio (HR) and the 95% CI were present. Schoenfeld’s partial residuals were utilized to test the proportionality assumption.

Subgroup analysis was conducted to compare the incidence of primary outcomes across different clinical conditions which can influence prognosis, and interaction analysis was conducted to examine the heterogeneity.

When p was <0.05, it was considered as statistically significant. Statistical analysis was carried out by using Stata ver. 13.1 (Stata Corp, College station, Texas).

## Supplementary information

Supplementary table 1,2 and Figure
