# Supplementary material for: Clopidogrel plus Aspirin Use is Associated with Worse Long-Term Outcomes, but Aspirin Use Alone is Safe in Patients with Vasospastic Angina: Results from the VA-Korea Registry, A Prospective Multi-Center Cohort
Source: Sci Rep. 2019 Nov 28;9:17783. doi: 10.1038/s41598-019-54390-w (PMC6883054; doi:10.1038/s41598-019-54390-w)

Clopidogrel plus Aspirin Use is Associated with Worse Long-Term Outcomes, but Aspirin Use Alone is Safe in Patients with Vasospastic Angina: Results from the VA-Korea Registry, A Prospective Multi-Center Cohort.

Seong-Sik Cho, MD[a]; Sang-Ho Jo, MD[b*];Seung Hwan Han, MD[c]; Kwan Yong Lee, MD[d]; Sung-Ho Her, MD[e]; Min-Ho Lee, MD[f]; Won-Woo Seo, MD[g]; Sung Eun Kim, MD[g]; Tae-Hyun Yang, MD[h]; Keun-Ho Park, MD[i]; Jung-Won Suh, MD[j]; Byoung-Kwon Lee, MD[k]; Seung-Woon, Rha, MD[l]; Hyeon-Cheol Gwon, MD[m]; Sang Hong Baek, MD[n]

[a]Department of Occupational and Environmental Medicine, College of Medicine, Dong-A University, Busan, Korea

[b]*Division of Cardiology, Department of Internal Medicine, Hallym University Sacred Heart Hospital, Anyang-si, Gyeonggi-do, Korea

[c]Department of Cardiovascular Medicine, Gil Medical Center, Gachon University, Incheon,

[d] Department of Cardiovascular Medicine, Incheon St. Mary's Hospital, The Catholic University of Korea, Incheon, South Korea

[e]Department of Cardiovascular Medicine, Daejeon St. Mary's Hospital, The Catholic University of Korea, Daejeon, South Korea

[f]Department of Cardiovascular Medicine, Soonchunhyang Seoul Hospital, Seoul, South Korea

[g]Department of Cardiovascular Medicine Hallym University Kangdong Hospital, Seoul, South Korea

[h]Department of Cardiovascular Medicine, Busan Paik Hospital, Inje University, Busan, South Korea

[i]Department of Cardiovascular Medicine, Chosun Medical Center, Gwangju, South Korea

[j]Department of Cardiovascular Medicine, Bundang Hospital, Seoul National University,

Seongnam, South Korea

[k]Department of Cardiovascular Medicine, Gangnam Severance Hospital, Yonsei University, Seoul, South Korea

[l]Department of Cardiovascular Medicine, Guro Hospital, Korea University, Seoul, South Korea

[m]Department of Cardiovascular Medicine, Samsung Medical Center, Sungkyunkwan University, Seoul, South Korea

[n]Department of Cardiovascular Medicine, Seoul St. Mary's Hospital, The Catholic University of Korea, Seoul, South Korea

Supplementary table 1. result of Cox proportional hazard regression for primary outcome additionally including athesclerosis and tropnin-I in the model (n=937)

	HR	95%CI		p
Age	1.00	0.98	1.02	0.928
Female	0.98	0.56	1.73	0.949
History of CHD	1.04	0.53	2.04	0.918
Smoking	1.20	0.69	2.10	0.523
Hypertension	0.86	0.50	1.48	0.583
DM	0.94	0.40	2.23	0.897
Dyslipidemia	1.75	1.01	3.05	0.048
Calcium Channel blocker	1.36	0.83	2.23	0.228
Definite spasm	1.12	0.45	2.81	0.812
Atherosclerosis ≥ 50%	1.07	0.44	2.59	0.886
Troponin-I	1.03	1.00	1.06	0.077
**Antiplatelet agents**				
No antiplatelet agent	reference			
Aspirin	0.83	0.48	1.45	0.514
Clopidogrel	0.50	0.12	2.11	0.345
Aspirin + clopidogrel	1.84	0.89	3.79	0.100

HR: Hazard ratio, CI: confidence interval, CHD: Coronary heart disease, DM: diabetes mellitus, primary outcome includes arrhythmia, acute coronary syndrome, and death

Supplementary table 2. result of Cox proportional hazard regression for primary outcome additionally including athesclerosis and tropnin-I in the model among patients without history of PCI (n= 912)

	HR	95%CI		p
Age	0.99	0.97	1.02	0.636
Female	0.92	0.51	1.65	0.781
History of CHD	0.80	0.34	1.89	0.610
Smoking	1.04	0.58	1.86	0.901
Hypertension	0.95	0.54	1.66	0.854
DM	1.12	0.47	2.64	0.804
Dyslipidemia	1.71	0.97	3.04	0.066
Calcium Channel blocker	1.06	0.42	2.66	0.908
Definite spasm	1.29	0.77	2.17	0.337
Atherosclerosis ≥ 50%	0.84	0.29	2.41	0.740
Troponin-I	1.02	0.99	1.06	0.209
**Antiplatelet agents**				
No antiplatelet agent	reference			
Aspirin	0.89	0.51	1.56	0.682
Clopidogrel	0.54	0.13	2.28	0.399
Aspirin + clopidogrel	1.65	0.71	3.84	0.243

PCI: percutaneous coronary intervention, HR: Hazard ratio, CI: confidence interval, CHD: Coronary heart disease, DM: diabetes mellitus, primary outcome includes arrhythmia, acute coronary syndrome, and death

Supplementary Figure

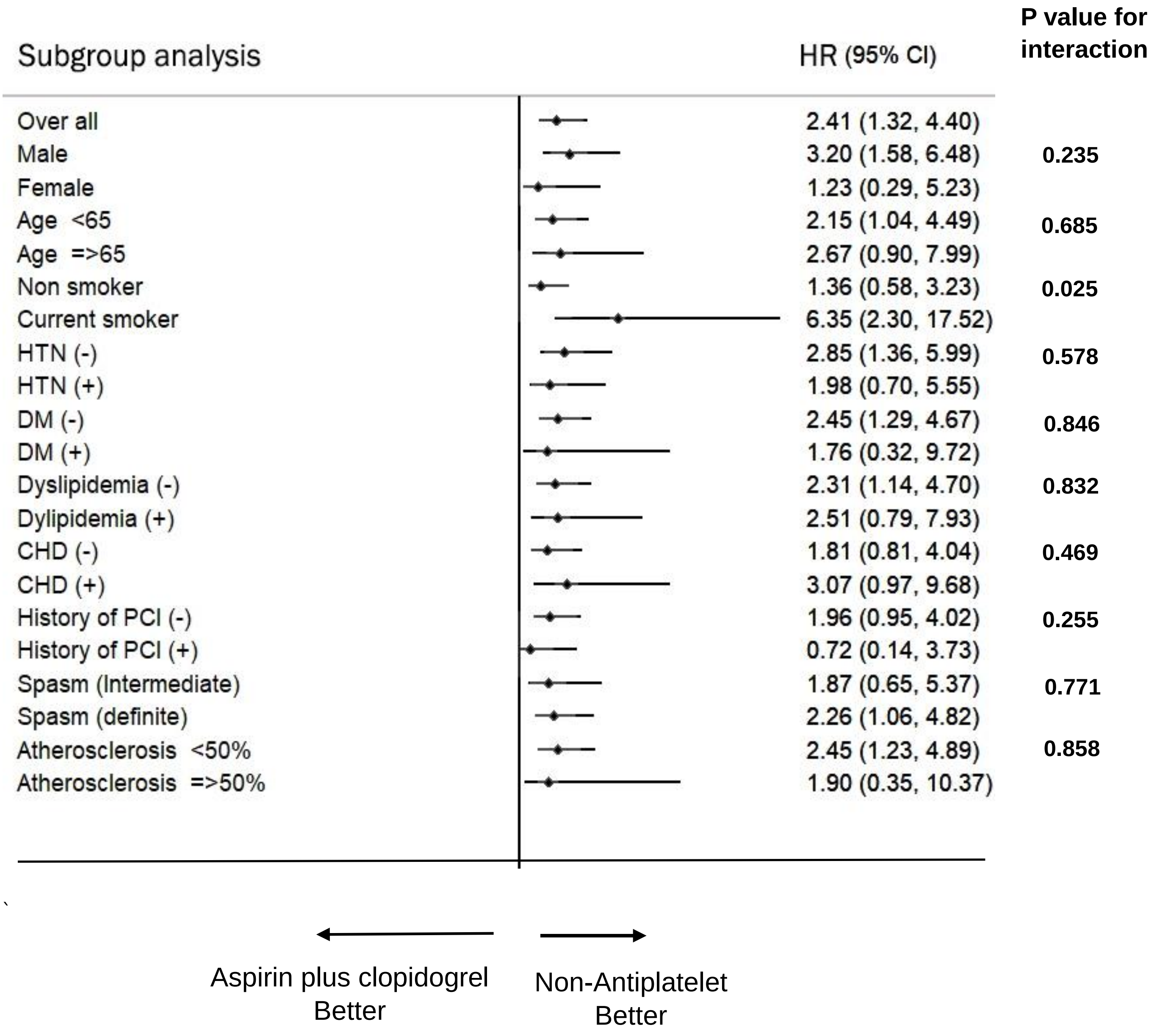

Supplement: Supplementary file 1 — Supplementary table 1,2 and Figure [file 41598_2019_54390_MOESM1_ESM.pdf]
